# Emergency Psychiatric Consultations During and After the COVID-19 Lockdown in Italy. A Multicentre Study

**DOI:** 10.3389/fpsyt.2021.697058

**Published:** 2021-06-15

**Authors:** Matteo Balestrieri, Paola Rucci, Davide Amendola, Miki Bonizzoni, Giancarlo Cerveri, Chiara Colli, Filippo Dragogna, Giuseppe Ducci, Maria Giuseppa Elmo, Lucio Ghio, Federico Grasso, Clara Locatelli, Claudio Mencacci, Leonardo Monaco, Alessandra Nicotra, Giulia Piccinini, Livia Pischiutta, Marco Toscano, Marco Vaggi, Vincenzo Villari, Alberto Vitalucci, Giulio Castelpietra, Emi Bondi

**Affiliations:** ^1^Unit of Psychiatry, Department of Medicine (DAME), University of Udine, Udine, Italy; ^2^Department of Biomedical and Neuromotor Sciences, Alma Mater Studiorum, University of Bologna, Bologna, Italy; ^3^Servizio Psichiatrico Diagnosi e Cura, Dipartimento Salute Mentale, Azienda Sanitaria Locale (ASL) Sa/2, Salerno, Italy; ^4^Department of Mental Health and Addiction, Azienda Socio-Sanitaria Territoriale (ASST) Lodi, Lodi, Italy; ^5^Department of Mental Health-Addiction and Neuroscience, Azienda Socio-Sanitaria Territoriale (ASST) Fbf-Sacco, Milan, Italy; ^6^Dipartimento Salute Mentale, Azienda Sanitaria Locale (ASL) Roma 1 – Serivzio Psichiatrico di Diagnosi e Cura (SPDC) Ospedale San Filippo Neri, Rome, Italy; ^7^Department of Mental Health and Addiction, Azienda Sanitaria Locale 3 (ASL3) Genova, Genova, Italy; ^8^Department of Psychiatry, Azienda Socio-Sanitaria Territoriale (ASST) Papa Giovanni XXIII, Bergamo, Italy; ^9^Azienda Socio-Sanitaria Territoriale (ASST) Rhodense, Garbagnate Milanese, Italy; ^10^Struttura Complessa (SC) Psichiatria Serivzio Psichiatrico di Diagnosi e Cura (SPDC), Dipartimento Neuroscienze e Salute Mentale, Azienda Ospedaliera Universitaria (AOU) Città della Salute e della Scienza, Torino, Italy; ^11^Outpatient and Inpatient Care Service, Central Health Directorate, Friuli Venezia Giulia Region, Trieste, Italy

**Keywords:** psychiatric consultations, emergency department, suicidal ideation, COVID-19, psychotropic drugs

## Abstract

**Aims:** The aim was to analyse the psychiatric consultations in nine Italian hospital emergency departments, by comparing the lockdown and post-lockdown periods of 2020 with the equivalent periods of 2019.

**Methods:** Characteristics of psychiatric consultations, patients, and drug prescriptions were analyzed. Joinpoint models were used to identify changes in the weekly trend of consultations.

**Results:** A 37.5% decrease in the number of consultations was seen during the lockdown period and 17.9% after the lockdown. The number of individual patients seen decreased by 34.9% during the lockdown and 11.2% after the lockdown. A significant change in the number of consultations from week 11 to week 18 occurred, followed by a gradual increase. There was a higher percentage of patients with previous psychiatric hospitalizations during the lockdown period (61.1 vs. 56.3%) and a lower percentage after the lockdown (59.7 vs. 64.7%). During the lockdown there was a large increase in psychiatric consultations for substance use disorders, whereas more consultations for manic episodes occurred after the lockdown. A 3.4% decrease was observed in consultations for suicidal ideation and planning during the lockdown, followed by an upward rebound after the lockdown, along with an increase in consultations for suicide attempts. During lockdown antipsychotic and benzodiazepine prescriptions increased by 5.2 and 4.1%, respectively. After the lockdown, the number of compulsory hospitalizations was higher than in 2019.

**Conclusions:** We observed a decrease of psychiatric consultations during and after the lockdown. There was an increase in consultations for manic episodes and suicidality after the lockdown. The focus of psychiatric services must remain high particularly in this latter period.

## Introduction

To date, much work has focused on medium-term mental health consequences of COVID-19 pandemic (1). In general, projections indicate an increase in depression and post-traumatic stress disorder (PTSD) on a basis of biological mechanisms and social consequences (2, 3). On the other hand, some preliminary reports on mental health management in the acute phase of the pandemic have found a decrease in requests for psychiatric consultations in hospital emergency departments (HEDs) (4–11). Clearly, the type of organization of mental health care has a strong influence on how each country copes with the crisis. Italy, which was the European country with the highest initial impact of the pandemic (12), has a community-based organization of mental health care. A nation-wide network of Mental Health Departments provides outpatient care primarily through Community Mental Health Centres (CMHCs), but also operates semi-residential and residential facilities. Inpatient care is provided through small General Hospital Psychiatric Units (GHPUs) with no more than 15 beds. In total, there are 1.7 acute care beds per 10,000 population, one of the lowest numbers in Europe (13). Some Italian regions have CMHCs operating 24 h a day. The law stipulated that GPHUs should have no more than 15 beds, in order to avoid the reproduction of large-scale, asylum-like wards. Public mental health care is typically provided free of charge to the entire population and is by far the most widely used form of mental health care in the country (14).

As a general rule, patients with acute psychiatric symptoms usually access directly hospital emergency departments (HEDs), accompanied by family members, or are referred by CMHCs, but they may also access the HEDs through emergency medical services, accompanied by the police in some cases. The HEDs then may require a psychiatric consultation to determine whether admission to GPHU is needed. Normally, patients are hospitalised in GHPU on a voluntary basis, but there are conditions requiring compulsory admission. These regard the urgent need of care for the patient, the patient's refusal to be treated, and no feasible outpatient treatment alternatives. Compulsory treatment procedure is then activated, in line with Italian law No. 833/1978.

In this context, the outbreak of COVID-19 pandemic has produced significant challenges on the mental health organization (15–18). On March 8, 2020, a government decree introduced in Italy a number of restrictions to counteract the spread of the pandemic. The national lockdown lasted from March 9 to May 17, 2020. At that time Italy was the European country with the highest incidence rate of COVID-19 cases. Official data provided by the Italian Civil Protection Department on March 9, 2020 reported 9,172 cases throughout the country with 463 deaths, while on May 18, 2020, at the end of the lockdown period, there were 225,886 cases and 32,007 deaths (19).

The government decree stated that during lockdown mental health departments had to maintain full functionality of mental health and substance abuse services. Moreover, they were required to organise emergency operation plans. Since the beginning of the lockdown, patients with psychiatric symptoms had to be screened for COVID-19 symptoms in HED before accessing GPHUs.

The aim of this study was to analyse the frequency and the characteristics of psychiatric consultations in HEDs in nine hospitals located in Northern, Central, and Southern Italy during the lockdown and post-lockdown periods of 2020, compared with those of the equivalent periods in 2019. We hypothesised that because the lockdown is a period of major reorganization in social and health care, there would be a reduction in HED psychiatric consultations during this period, mostly targeted to patients with severe psychopathology. We also hypothesised that the number of psychiatric consultations would increase in the post-lockdown period of 2020 because of the gradual return to normalcy.

## Materials and Methods

This study was conducted on behalf of the National Coordination of Italian Psychiatric Diagnostic and Treatment Services, section of the Italian Society of Psychiatry, and it was approved by the ethics committees of Bergamo (Reg. Sperim. N.260/20) and Udine (CEUR-2021-OS-05), Italy. Information on HED psychiatric consultations of patients was retrospectively collected between March 9, 2020 and June 30, 2020 and during the same period of the previous year from patient records in 9 Italian centres, 4 of which were in Lombardy Region, that was the first hit by COVID-19 pandemic (59.6% of cases nationwide on March 9, 2020). The period between 8 March and 17 May 2020 was considered the lockdown period, while the period between 18 May and 30 June 2020 was considered the post-lockdown period.

Data were retrieved from the clinical administrative databases of the Health Agencies and analysed using an anonymous patient identifier, in accordance with Data Protection Act (EU Regulation 679/2016). Diagnoses were assigned on a clinical basis and coded at each centre according to ICD 9-CM criteria. The demographic and clinical characteristics of the study sample were summarised using mean and standard deviation or absolute and percentage frequencies.

We analysed the trends of HED psychiatric consultations during the lockdown and the post-lockdown periods and compared them with those of the equivalent periods of the previous year. Joinpoint models were used to identify changes in the slopes of weekly psychiatric consultations within the same year and between years. This was done by fitting trend data with a 0 joinpoint model, which is a straight line, and then testing whether more joinpoints were statistically significant and had to be added to the model. We modelled the weekly counts of HED psychiatric consultations as a function of the week using a Poisson model of variation. The significance of the percentage rate changes within the observation periods was tested using a Monte Carlo Permutation method. The joinpoint regression curves of HED psychiatric consultations were compared between the two periods using the parallelism test, that tests whether two joinpoint regression functions are parallel. When the null hypothesis of parallelism is rejected, there is an indication that regression curves change their slope at different time points during the observation period. Statistical analyses were carried out using IBM SPSS, version 25.0. The trend of HED psychiatric consultations was analysed using the Joinpoint Trend Analysis Software 4.8.0.1 (Statistical Research and Applications Branch, National Cancer Institute, USA).

## Results

The number of patients seen in HED for psychiatric consultations decreased from 2639 in 2019 to 1954 in 2020, with a 34.9% reduction during the lockdown and 11.2% reduction after the lockdown. No difference in the demographic characteristics and clinical history of patients was found in the lockdown and the post-lockdown periods compared with the corresponding periods of 2019 (Table 1), except for a higher percentage of patients with previous admissions to GPHUs during the lockdown period (61.1 vs. 56.3%; *p* = 0.019). In the post-lockdown period, these percentages reversed, so that a lower proportion of patients with previous hospitalizations were seen in the HEDs in 2020 than in 2019 (59.7 vs. 64.7%; *p* = 0.034).

**Table 1:** Patient characteristics by period and year.

		**Lockdown period**					**Post-lockdown period**				
		**2019 (No. 1,649)**		**2020 (No. 1,075)**		***p*-value**	**2019 (No. 990)**		**2020 (No. 879)**		***p*-value**
Current age (years), *mean±SD*		44.7	17.1	45.5	17.6	0.185	44.2	17.3	44.6	17.2	0.611
Gender, *No. and %*	Female	786	50.1%	499	48.3%	0.392	499	50.4%	433	49.3%	0.622
	Male	783	49.9%	535	51.7%		491	49.6%	446	50.7%	
Marital status, *No. and %*	Married/living with partner	288	18.4%	226	21.8%	0.119	206	20.8%	180	20.5%	0.842
	Unknown	525	33.5%	281	27.1%		279	28.2%	209	23.8%	
	Single	592	37.7%	422	40.8%		402	40.6%	384	43.7%	
	Separated/Divorced	154	9.8%	104	10.0%		98	9.9%	100	11.4%	
	Widow	10	0.6%	2	0.2%		5	0.5%	5	0.6%	
Working status, *No. and %*	Never employed	212	21.4%	154	22.4%	0.941	143	22.3%	157	25.8%	0.751
	Recently lost job	72	7.3%	52	7.5%		43	6.7%	38	6.3%	
	Employed	238	24.0%	164	23.8%		168	26.3%	151	24.8%	
	Retired	142	14.3%	89	12.9%		90	14.1%	88	14.5%	
	Disability pension	185	18.7%	125	18.1%		97	15.2%	91	15.0%	
	Other	142	14.3%	105	15.2%		99	15.5%	83	13.7%	
Previous admissions to GHPU, *No. and %*	No	779	56.3%	611	61.1%	0.019	578	64.7%	468	59.7%	0.034
	Yes	605	43.7%	389	38.9%		315	35.3%	316	40.3%	

As shown in Table 2, overall 3,185 HED psychiatric consultations, including multiple consultations to the same patients, were recorded in the year 2019 and 2,237 in the year 2020, that amounts to a 29.7% reduction (−37.5% during the lockdown and −17.9% after the lockdown). A decrease in consultations during the lockdown occurred in all centres except for Udine. On the other hand, during the post-lockdown period, the number of HED psychiatric consultations returned to values between +/−15% of those in 2019 in six out of nine centres.

**Table 2:** Psychiatric and overall consultations by period, year and centres.

	**Psychiatric consultations in HED**									**Overall admissions in HED**		
		**Lockdown period**			**Post-lockdown period**			**Full period**			**Full period**		
		**2019**	**2020**		**2019**	**2020**		**2019**	**2020**		**2019**	**2020**	
		**No**.	**No**.	**%change**	**No**.	**No**.	**%change**	**No**.	**No**.	**%change**	**No**.	**No**.	**%change**
Centre	Bergamo	360	283	−21.4	237	225	−5.1	597	508	−14.9	21,653	16,739	−22.7
	Codogno	74	36	−51.4	47	42	−10.6	121	78	−35.5	8,429	4,467	−47.0
	Milano Fatebenefratelli	362	220	−39.2	191	207	8.4	553	427	−22.8	15,990	7,225	−54.8
	Garbagnate	326	195	−40.2	208	178	−14.4	534	373	−30.1	38,517	16,849	−56.3
	**Lombardy**	**1,122**	**734**	**−34.6**	**683**	**652**	**−4.5**	**1,805**	**1,386**	**−23.2**	**84,589**	**45,280**	**−46.5**
	Torino	209	121	−42.1	155	51	−67.1	364	172	−52.7	22,050	19,860	−9.9
	Genova DSM	223	66	−70.4	128	86	−32.8	351	152	−56.7	9,137	4,128	−54.8
	Udine	38	41	7.9	49	50	2.0	87	91	4.6	2,347	2,090	−11.0
	Roma	270	194	−28.1	194	152	−21.6	464	346	−25.4	7,619	4,858	−36.2
	Salerno	69	51	−26.1	45	39	−13.3	114	90	−21.1	402	270	−32.8
	**Other regions**	**809**	**473**	**−41.5**	**571**	**378**	**−33.8**	**1,380**	**851**	**−38.3**	**41,555**	**31,206**	**−24.9**
	**All Centres**	**1,931**	**1,207**	**−37.5**	**1,254**	**1,030**	**−17.9**	**3,185**	**2237**	**−29.8**	**126,144**	**76,486**	**−39.4**

*Values in bold are the summary values of the centres listed above*.

The overall number of HED admissions for any type of health problems is reported for comparison in the last two columns of Table 2. A 39.4% reduction was found in the pooled data of the 9 centres, that varied from 46.5% in Lombardy, the region most heavily affected by COVID-19 disease, to 24.9% in the other Italian centres. Notably, the proportion of psychiatric HED consultations over all HED admissions slightly increased between 2019 and 2020 (2.5 vs. 2.9%).

No difference was found in the proportion of consultations that led to GPHU admissions or compulsory admissions during the lockdown period (Table 3). In contrast, in the post-lockdown period, GPHU admissions and the number of compulsory admissions were substantially higher than in the equivalent period of 2019.

**Table 3:** Characteristics of psychiatric consultations by period and year.

		**Lockdown period**					**Post-lockdown period**				
		**2019**		**2020**			**2019**		**2020**		
		**No**.	**%**	**No**.	**%**	***p*-value**	**No**.	**%**	**No**.	**%**	***p*-value**
Admitted to GHPU		740	38.3%	441	36.6%	0.318	465	37.1%	513	49.8%	<0.001
Compulsory admission		109	5.6%	87	7.2%	0.075	67	5.3%	82	8.0%	0.011
Diagnosis made by the psychiatrist						<0.001					0.001
	No psychiatric disorder	26	1.3%	31	2.6%	*	15	1.2%	17	1.7%	
	Psychotic episode	241	12.5%	143	11.9%		155	12.4%	136	13.3%	
	(Hypo)manic agitation	95	4.9%	36	3.0%	*	48	3.8%	57	5.6%	*
	Psychomotor agitation	449	23.3%	287	23.8%		316	25.2%	241	23.5%	
	Depression	205	10.6%	81	6.7%	*	112	8.9%	94	9.2%	
	Anxiety	384	19.9%	262	21.7%		255	20.3%	149	14.5%	*
	Adjustment disorder/Distress	76	3.9%	30	2.5%	*	53	4.2%	42	4.1%	
	Suicidal ideation/attempt	211	10.9%	133	11.0%		123	9.8%	131	12.8%	*
	Substance use disorders	133	6.9%	111	9.2%	*	113	9.0%	88	8.6%	
	Delirium	42	2.2%	24	2.0%		29	2.3%	19	1.9%	
	Other	69	3.6%	67	5.6%	*	35	2.8%	51	5.0%	*
Suicidality						0.004					0.003
	Absent/non-detectable	1502	83.2%	1046	86.7%	*	944	85.0%	739	79.3%	*
	Ideation or plans	179	9.9%	78	6.5%	*	91	8.2%	108	11.6%	*
	Suicide attempt	124	6.9%	83	6.9%		75	6.8%	85	9.1%	*
Pharmacological treatment											
	Neuroleptics	240	12.4%	212	17.6%	<0.001	170	13.6%	180	17.5%	0.010
	Lithium/mood stabilizers	165	8.5%	106	8.8%	0.818	102	8.1%	93	9.0%	0.446
	Antidepressants	211	10.9%	94	7.8%	0.004	135	10.8%	112	10.9%	0.934
	Benzodiazepines	665	34.4%	465	38.5%	0.020	416	33.2%	421	40.9%	<0.001
	Ketamine/propofol/ midazolam	108	5.6%	52	4.3%	0.111	70	5.6%	42	4.1%	0.098

* *Significant post-hoc pairwise comparisons*.

**Figure 1:**
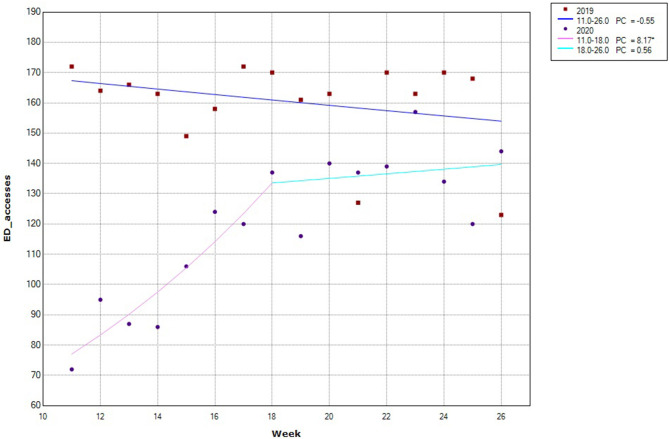
Trend of HED consultations during the study period in 2019 and 2020. PC = weekly percentage change. *significant change. In 2019, no significant change in slope was observed, while in 2020 one change in slope was observed at week 18 (starting from April 29th), when consultations increased at a slower pace compared to the previous period.

Figure 1 shows the weekly trend of consultations during the observation period in 2020 and the comparison period in 2019. A large discrepancy can be observed in the number of consultations between the 2 years, which decreased over time but did not completely cancel out. As to the annual trend in 2020, a significant percentage change in the number of consultations occurred from week 11 (March 11–17) to week 18 (April 29–May 5), followed by a more gradual, non-significant increase. Conversely, the trend in 2019 was stable over time, with a slight, non-significant decline.

As for diagnoses made during psychiatric consultation, during the lockdown there was a decrease manic episodes, depression and adjustment disorders but a sharp increase of substance use disorders compared with the equivalent 2019 period. In the 2020 post-lockdown period there was a higher proportion of consultations for manic episodes and suicidal ideation or attempts compared with 2019. On the contrary, the proportion of consultations for anxiety disorders was lower than in 2019 (Table 3).

When considering suicidality, the 2 years showed a significant difference both during the lockdown and in the post-lockdown periods. Most notable is the sharp decline (−3.4%) in consultations for suicidal ideation and planning during the 2020 lockdown (Table 3), followed by an upward rebound of the same in the post-lockdown (+3.4%) accompanied by an increase in consultations for suicide attempts (+2.3%).

Table 3 also shows an increase during the lockdown in the prescriptions of antipsychotics (+5.2%), benzodiazepines (+4.1%) and a decrease in antidepressants (−3.1%). In the post-lockdown, the increase in antipsychotics and benzodiazepines prescriptions continued (+3.9 and 7.7%, respectively), while antidepressant prescriptions were comparable to the previous year.

## Discussion

To our knowledge, this is the first Italian national-level study to investigate the impact of pandemic COVID-19 on psychiatric consultations in emergency departments. In Italy, few studies have focused on the organizational aspects related to the emergency, describing the changes that occurred to curb the difficulty of managing psychiatric patients and caregivers (16, 18, 20, 21). Early data from mental health departments in Lombardy during the lockdown were also analysed with great timing (22–24), suggesting a substantial decrease in psychiatric consultations. The decrease involved all diagnostic categories except for personality disorders, alcohol, substance abuse disorders and trauma- and stressor-related disorders. Similarly, in a study carried out in Emilia Romagna Region a 15% reduction was found in psychiatric referrals to the HEDs from the beginning of the lockdown to the 3rd of May 2020, which was almost doubled during the first month (25).

The present study aimed to analyse the effects of the lockdown measures adopted to slow down the spread of the SARS-CoV-2 infection on psychiatric emergencies in nine areas of different Italian regions, differing in the time and the extent of spread of the epidemic (particularly high in Lombardy). The effects detected can be interpreted both as a possible shaping of human behaviour in emergency conditions and an effect of the increasing difficulty in accessing HEDs. In this respect, however, it must be emphasised that HEDs have always remained open even under conditions of severe pressure. With a few exceptions that do not relate to the centres examined, a filtering of access certainly did not occur because of the closure of HEDs, but rather because of fears of moving out of one's home to reach the hospital or fear of getting in touch with SARS-CoV-2 positive persons.

Another useful aspect we can glean from our study is the observation of what happened after the lockdown. Even at its end, the number of consultations never returned to the level of the year 2019, suggesting that people in need of care postponed the HED visits.

The main findings of this epidemiological study can be summarised as follows. Over the period examined, there was a 30% reduction in HED psychiatric consultations in 2020 compared with 2019. This reduction in psychiatric consultations in the early stages of the pandemic is consistent with evidence from other countries (4–11).

Although the reduction was much higher in the lockdown period, there was a shrinkage of consultations in the following period as well. The drop in consultations during the lockdown was almost generalised (with one exception), but there were marked differences among participating centres. Notably, no differences were found between the centres in Lombardy, where the impact of the pandemic was particularly strong, and other centres. This suggests that the observed trend was more related to lockdown than to the degree of spread of the pandemic. On the other hand, during the lockdown in the Lombardy centres, the reduction in psychiatric consultations was half of the reduction in total consultations. This may indicate that psychiatric emergencies can be reduced under conditions of severe social crisis, but not beyond a certain limit, whereas other medical emergencies can do so to a greater extent.

Another interesting aspect is that the decrease in consultations was not due to a selection of patients with specific socio-demographic characteristics. The only significant difference was that more patients with previous GPHU admissions accessed the HEDs during the 2020 lockdown. Possible interpretations of this results are the selection of more severe patients or CMHC difficulties in managing acute cases because of a shortage of operators or difficulties in seeing patients at home, or even a combination of these.

In contrast, when comparing the two post-lockdown equivalent periods, a lower proportion of patients with previous hospitalizations was found in 2020, but a higher number of admissions, including compulsory admissions, to GPHUs. That is, more people were admitted in 2020, with more severe conditions, but with more recent onset of the disorder.

If we analyse the diagnostic distribution at HED admission over the 2 years, during the lockdown fewer diagnoses of manic episodes, depression, and adjustment disorders were made, while the diagnosis of substance use disorders was made more frequently. The latter can be a result of organizational difficulties in addiction services, which are traditionally more fragile and exposed to staff shortages under stressful conditions. On the other hand, in the post-lockdown fewer diagnoses of anxiety episodes were made, but a higher percentage diagnoses of manic episodes and suicidal ideation or attempts. In a comparison between consecutive periods of 2020, Ferrando et al. (4) found an increase in manic disorders and psychoses and less suicide attempts after the COVID-19 outbreak. In our study, we found a significant decline in suicidal ideation and planning during lockdown recorded during the psychiatric consultation, in line with Dvorak, followed by an increase in suicidal ideation, planning, and attempts in the subsequent phase. In a prospective cohort monitored using smartphone-delivered assessment, Cobo et al., observed that self-reported suicide risk decreased during the COVID-19-related lockdown period (26). These data corroborate historical data indicating a decrease in suicides under conditions of severe social distress, such as wars, and early published data on psychiatric emergencies during the 2020 pandemic (4, 27–29).

Lastly, our results concerning the prescription of drugs indicate an increasing need to treat anxiety and psychotic symptoms with drugs with sedative action on acute symptoms starting from the lockdown period.

### Limitations and Strengths

We note two limitations of our findings. First, data refer only to the first wave of the pandemic and subsequent changes in the HED consultations during the second and the third wave may add important insights. Second, although the study was conducted on behalf of the National Coordination of Italian Psychiatric Diagnostic and Treatment Services, the number of centres involved is relatively small, because of limitations in data availability or difficulties in extracting and harmonizing data from the local administrative databases.

One important strength is that we focused on the same time frames in 2019 and 2020, and analysed the same variables, thereby eliminating possible biases resulting from seasonality and data collection methodology.

In summary, our findings underscore a marked decline in consultations during the lockdown and a selection of patients with more severe psychiatric history. The overall distribution of diagnoses showed an increase in substance use disorders and a decrease of suicidality. In the post-lockdown, a higher percentage of patients without prior hospitalization had to be admitted, including mandatory admissions, than in 2019. Consultations for suicidal ideation, planning, and attempts increased sharply during this period, as did manic episodes. Antipsychotic medications and benzodiazepines were more frequently prescribed than in the previous year, reflecting the need for pharmacological management of anxiety and psychomotor agitation. Given the observed increase in consultations for manic episodes and suicidality at the end of the restriction period, the attention of mental health services to severe manifestations of the disorders should remain high.

### Implications for Research and Clinical Practice

Our experience in the conduction of this study points to the need to plan on a routine basis the timely collection and analysis of data concerning psychiatric emergencies in a structured way to enable comparisons among HEDs within and between regions for research purposes and to inform decisions at local and national level.

As to clinical practice, our findings underscore the need to facilitate access to psychiatric care for those in need, as well as implementing alternative outreach strategies to be prepared for possible critical events in the future. Such strategies may include telepsychiatry services to support patients to cope with isolation, feelings of loneliness, hopelessness, and helplessness that are associated with suicidality and might be amplified during health emergencies and imposed social distancing measures.

## Data Availability Statement

The raw data supporting the conclusions of this article will be made available by the authors, without undue reservation.

## Ethics Statement

The studies involving human participants were reviewed and approved by Ethics committees of Bergamo (Reg. Sperim. N.260/20) and Udine (CEUR-2021-OS-05), Italy. Written informed consent for participation was not required for this study in accordance with the national legislation and the institutional requirements.

## Author Contributions

MaB and EB contributed to conception and design of the study. CC, AN, and LP organized the database. DA, MiB, GC, FD, GD, ME, LG, FG, CL, CM, LM, GP, MT, MV, VV, AV, and EB collected the data. PR performed the statistical analysis. MaB wrote the first draft of the manuscript. MaB, PR, and GC contributed to the final draft of the manuscript. All authors contributed to the article and approved the submitted version.

## Conflict of Interest

The authors declare that the research was conducted in the absence of any commercial or financial relationships that could be construed as a potential conflict of interest.
